# Investigation of Microwave Electromagnetic Fields in Open and Shielded Areas and Their Possible Effects on Biological Structure

**DOI:** 10.3390/s23042351

**Published:** 2023-02-20

**Authors:** Filip Vaverka, Milan Smetana, Daniela Gombarska, Zuzana Psenakova

**Affiliations:** Department of Electromagnetic and Biomedical Engineering, Faculty of Electrical Engineering and Information Technology, University of Zilina, Univerzitna 8215/1, 010 26 Zilina, Slovakia

**Keywords:** microwave electromagnetic field, biological effects, human head phantom, shielded space, hygiene standards

## Abstract

The article’s subject is the investigation of electromagnetic fields (EMF) of the microwave frequency band in a typical human living environment, especially in shielded areas. The point of view of electromagnetic field presence in the environment with the rapid increase in the level of the electromagnetic background is currently an essential point concerning population protection against the potential adverse effects of such EMFs. The authors focus on actual measurements, especially in shielded spaces frequently used in everyday life, such as elevator cabins and cars. The goal is a quantitative evaluation of the distribution of specific vector quantities of the EM field and a comparison with the currently valid hygiene standards. Measured values in shielded spaces show elevated levels in contrast to the open space. However, the values do not exceed limits set by considering the thermal effect on living tissues.

## 1. Introduction

Living organisms have been exposed to natural electromagnetic radiation for about 4 billion years of Earth’s existence. The nature of life itself does require the EMF for its existence. Yet, the level of EMF radiation from natural sources has recently been surpassed by that of artificial sources. In fewer than 20 years, the frequencies and intensities of the diverse spectrum of artificial sources have likewise increased rapidly. Artificial EMFs are all around us, which raises concerns about how they affect human health and wellbeing of other living things as well. The paper aims to measure the mobile technologies’ microwave frequency range (GSM, Wi-Fi, Bluetooth, LTE, UMTS, etc.) in open and shielded areas with focus of shielded areas. Electromagnetic fields above certain levels can cause biological effects, which may be positive or negative. Scientific studies and research indicate that exposure to EMF at levels naturally present in the environment does not cause apparent adverse effects. National and international guidelines limit exposure to higher levels of EMF that may be harmful. The research worldwide is still focused on confirming or renouncing whether long-term exposure to artificial EMFs can produce biological effects and affect human health and to what extent this is possible. Increasing the temperature of human body tissues is the main considered biological effect of non-ionizing radiation, originating mainly in radio frequency fields. The radio frequency EMF levels that people are typically exposed to are much lower than those required to produce a thermal effect that would significantly affect the human body. The thermal effect of radio waves forms the basis for the current guidelines.

The transmission of information using electromagnetic waves from one place to another without cables or other electrical conductors is generally referred to as wireless communication. Wi-Fi allows the devices to exchange information with each other and create a network. Internet connection takes place using a wireless router. Wireless communication devices are a necessity in everyday life these days. With the help of a mobile phone and the Internet, we can stay in touch even over long distances. The Internet of Things allows us to communicate with the machines in our homes. It may make our day-to-day life easier, but the effect of exposure to a high-frequency EM field is inevitable for everyone, from newborns to seniors. These devices can offer significant benefits, but on the other hand, the high-frequency radiation emanating from the device is not natural to the human living environment. It may represent a potential health risk.

Both objective and subjective parameters are connected to the biological impacts of EM radiation. Utilized frequency range, intensity, field type, and exposure time are examples of physical parameters. Dimensions, weight, the surface material of the clothing, the thickness of tissue layers (skin, fat, muscles), percentage of body water, state of health, and potential effects of other factors such as stress, age, diseases, etc. are all aspects of physical and chemical parameters. The effects of the electromagnetic field (EMF) on a person can be classified as thermal effects (manifest at high field intensities) and non-thermal effects (specific) (expressed by altering the colloidal structure and electrical properties of the cell).

The World Health Organization (WHO) has included mobile phone radiation to have a possible carcinogenic effect. Studies conducted at several medical, pharmaceutical, and technical universities concluded that the influence of mobile phones leads to eye strain, lack of sleep, migraines, restlessness, and irritability and may cause hearing impairment. Radiation from mobile phones and their effect has been found to have a greater effect on people under 20 years of age [1,2,3,4,5,6,7,8].

The International Commission on Non-Ionizing Radiation Protection (ICNIRP) has established electromagnetic field exposure limits for human safety. Temperature elevation is the ICNIRP-identified mechanism of relevance for the frequencies used, and restrictions are set to avoid a ‘significant’ increase in temperature. ICNIRP differentiates between steady-state temperature rises (where temperature increases slowly, allowing time for heat to dissipate and for body thermoregulatory processes to take effect) and brief temperature rises (where there may not be sufficient time for heat to dissipate). The temperature rise thresholds for steady-state exposures are listed in Table 1 and summarized below.

The specific absorbed rate—*SAR* value expresses the degree to which biological tissue absorbs the energy of high-frequency or radio-frequency radiation. *SAR* is defined as the absorbed power in tissue weight.

**Figure 1:**
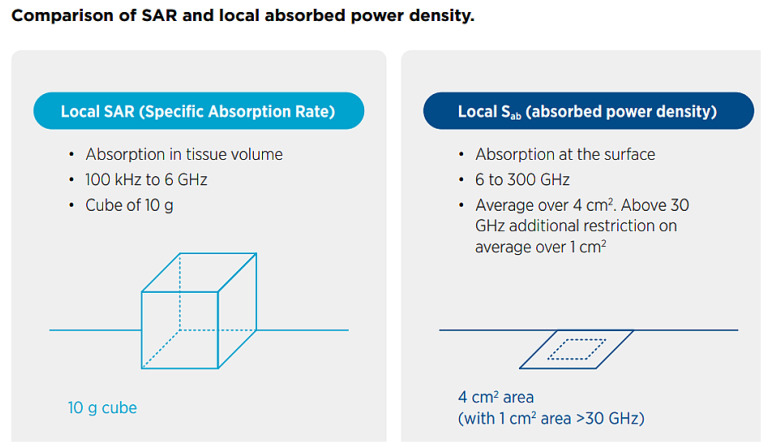
ICNIRP Comparison of local *SAR* and local absorbed power density *S*_ab_ [9].

The averaging time for whole-body exposure was changed to 30-min to account for the time it takes the whole body to reach steady-state temperature. The averaging time for both *SAR* and absorbed power density *S*_ab_ (Figure 1) was kept at 6 min as it closely matches the thermal time constant for local exposure [9].

**Figure 2:**
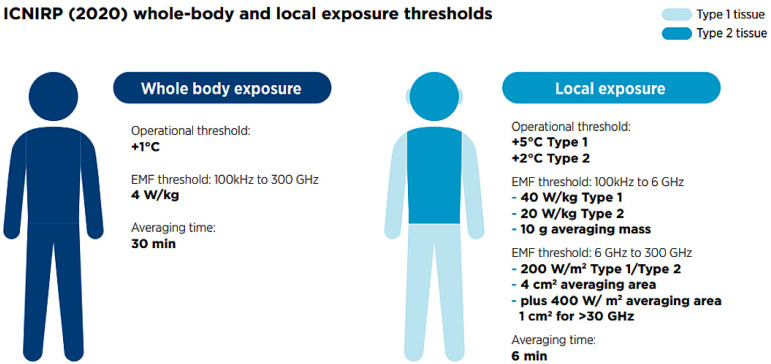
ICNIRP Whole- body and local exposure thresholds [9].

These limits, also for wireless communication devices, were created based on the performance of individual wireless devices in an open space for a period of 6 min, Figure 2. However, in shielded spaces the effect of electromagnetic radiation is stronger than outside the niche. The outer walls of the space, which often contain metal components, represent an obstacle for the EMF, where part of the EMF is reflected, part passes through, part is attenuated—in the extreme case it does not pass into the external environment, or accumulates and increases mainly inside the shielded space (because the source the EMF, which can be, for example, a mobile phone, tries to connect with the base station, and at the same time, since it is in a shielded space, it increases its power). Humans are commonly present in shielded spaces, whether they are cellar spaces in family homes, railway carriages, elevators, cars, or airplane cabins, which generally support the reflection and resonance effects of electromagnetic fields. As already mentioned above, a higher intensity of the electric field can occur in these areas, which can also represent a higher risk of endangering human health. The aim of this paper is to investigate these effects. We focus on the distribution of electric field strength using a mobile phone in the RF band in shielded areas. In the theoretical part of our work discuss the interaction and propagation of the electromagnetic field through the biological environment. The practical part deals with the distribution of electric field intensity in elevator cabin, and inside the car.

### Propagation of EM Waves and Its Interaction with Matter

The energy of the electromagnetic field spreads in space in the form of waves whose properties depend on the nature of the source and the nature of the transmission environment. In the simplest case, a plane electromagnetic wave is characterized by the fact that the quantities **E** and **H** are functions of one spatial coordinate of the Cartesian coordinate system. For example, from the *z* coordinate, these quantities are constant concerning the other two coordinates. It is possible to observe this case of a wave at a sufficient distance from the antenna when each wave is practically planar. If z denotes the direction of wave propagation, all quantities characterizing the field depend only on the coordinate z and time t, so it applies $E=E\left(z,t\right),\;H=H\left(z,t\right).$ The properties of the environment characterize its permittivity and permeability or the wave propagation speed *v* and wave impedance *Z*_0_. For an ideal dielectric, both quantities are frequency independent. The wave impedance *Z*_0_ of the lossless environment has a resistive character, so the wave components are in phase, Equation (1). It is frequency independent.

(1) $$Z_{0}=\frac{E_{m}}{H_{m}}=\sqrt{\frac{\mu }{\varepsilon }}\;,$$

As in the case of a dielectric medium, and also for a conductive medium, wave equations are established for any waveform and any coordinate system.

(2) $$\nabla^{2}H=\mu \gamma \frac{\partial H}{\partial t},\nabla^{2}E=\mu \gamma \frac{\partial E}{\partial t},$$

The symbolic-complex method for the harmonic course of the wave can be used

(3) $$\dot{E}\left(r,t\right)={\dot{E}}_{m}\left(r\right)\cdot e^{j\omega t},\;\;\;\;\;\;\;\;\;\dot{H}\left(r,t\right)={\dot{H}}_{m}\left(r\right)\cdot e^{j\omega t},$$

where the quantities ${\dot{E}}_{m}\left(r\right)\;\mathrm{and}\;{\dot{H}}_{m}\left(r\right)\;$ are complex maximum values, depending on the guide *r*. After substituting these Equation (3) into the wave Equation (2), time-independent harmonic wave equations for a conductive medium are

(4) $$\nabla^{2}\dot{H}=j\omega \mu \gamma {\dot{H}}_{m},\;\;\;\;\;\;\;\;\;\;\;\;\;\;\;\;\;\;\;\;\nabla^{2}\dot{E}=j\omega \mu \gamma {\dot{E}}_{m},$$

If considering the propagation of the wave in the direction of the z-axis, it is necessary to write these equations in the Cartesian coordinate system, and the partial derivatives according to the x and y coordinates will be equal to zero.

(5) $$\frac{d^{2}{\dot{H}}_{m}}{dz^{2}}=j\omega \mu \gamma \;\;\;\;\;\;\;\;\;\;\;\;\;\;\;\;{\dot{H}}_{m}\frac{d^{2}{\dot{E}}_{m}}{dz^{2}}=j\omega \mu \gamma {\dot{E}}_{m},$$

Equation (5) is used to solve the case when a wave from a lossless environment, a dielectric, penetrates a conductive, lossy environment. By solving these equations, we obtain the instantaneous values of the quantities ***H***(*z*,*t*) and ***E***(*z*,*t*). When further considering the relation ***E*** = ***J***/γ an instantaneous value for the quantity ***J***(*z*,*t*) is obtained.

(6) $$H_{y}=H\left(z,t\right)=H_{m0}\cdot e^{-kz}\cos\left(\omega t+\psi_{H0}-kz\right),\;E_{x}=E\left(z,t\right)=\sqrt{\frac{\omega \mu }{\gamma }}H_{m0}\cdot e^{-kz}\cos\left(\omega t+\psi_{H0}+45{}^{\circ}-kz\right),\;J_{x}=J\left(z,t\right)=\sqrt{\omega \gamma \mu }\cdot e^{-kz}\cos\left(\omega t+\psi_{H0}+45{}^{\circ}-kz\right),$$

where $k=\sqrt{\frac{µ\omega \gamma }{2}}.$

These three Equations (6) are expressed using the modulus and argument of the magnetic intensity at the dielectric–conductor interface. All quantities change in time at every point of the 𝑧 axis harmonically, and their amplitude gradually decreases exponentially with the increasing value of the coordinate 𝑧. Quantities **𝐸** and **𝐽** are in phase and shifted by 45° relative to **𝐻**, 𝜓**_𝐻_**_0_ is the initial phase at the interface between the dielectric and the conductor. In a situation where a harmonic plane wave passes through a dielectric medium and subsequently hits the interface of a conductive material, the EMF wave is reflected at an angle of reflection equal to the angle of incidence. The wave penetrates the conductive medium, where the wave propagates perpendicular to the interface, and damping occurs with an effective depth of penetration δ. When the wave hits the surface of the conductive material, there is practically a complete reflection.

Some energy is transmitted through the EM wave. The magnitude and direction of the flow of this energy are indicated by a vector quantity—the Poynting vector. We determine the corresponding energy density of the EM field by

(7) $$w=w_{e}+w_{m}=\frac{1}{2}\varepsilon E^{2}+\frac{1}{2}µH^{2}=\frac{1}{v}E\cdot H.$$

The energy density is inversely proportional to the velocity of propagation and directly proportional to the product of ***E***.***H***. Next, let us imagine the differential surface *ds*, which is located perpendicular to the direction of wave propagation. We determine the energy *dW* that passes through the area *ds* in time *dt*, while *dV* = *v*.*dt*.*ds* applies.

(8) dW=w·dV=E·H·dt·ds.

where *dW* is the differential flux of EM energy through the area *ds*, *dW*/d*s* are the flux density of EM energy and *dW*/*ds*·*dt* = ***E***·***H*** = *S*. Here, *S* is the power of the energy flow density, equal to the modulus of the Poynting vector *I*. The direction of energy propagation is identical to the direction of wave propagation. This direction is perpendicular to the plane where ***E*** and ***H*** lie, and we can introduce a vector quantity Poynting vector ***I*** = ***E*** × ***H***. The physical meaning of this vector is: the modulus of the vector ***S*** is equal to the power passing through the unit area perpendicular to the direction of wave propagation, and the direction of the Poynting vector is the same as the direction of wave propagation. If the surface is not perpendicular to the direction of propagation and is not even flat, the following relation applies:

(9) $$I=\int_{s}S\cdot ds=\int_{s}\left(E\times H\right)\cdot ds.$$

It follows from this description that the EM field is a carrier of energy with density ***w***, this energy is transmitted through waves. The vectors of quantities ***E*** and ***H*** are functions of time, so the Poynting vector is also time dependent. The intensity of electromagnetic radiation is defined as the mean value of the magnitude of the Poynting vector.

(10) I=⟨|E×H|⟩.

EMF can penetrate the human body and spread through it in the form of a wave. Tissue properties affect the field strength, wavelength, velocity of propagation, direction of propagation, temporal and spatial arrangement, and polarization of the propagating wave within the tissue. The total amount of absorbed energy is determined by the properties of the tissue. The penetration of the electromagnetic wave into the lossy environment is assessed according to the penetration depth.

In the dosimetry of non-ionizing electromagnetic radiation for the frequency interval from 100 kHz to 6 GHz, the introduced quantity is the specific absorbed rate *SAR* (W/kg). It is a quantity that characterizes the transfer of energy of electric and magnetic fields into a substance or its charged particles. The European Committee for Electro-technical Standardization (CENELEC) defines *SAR* as: “The time derivative of the incremental energy (*dW*) absorbed by (dissipated in) an incremental mass (*dm*) contained in a volume element (*dV*) of given density (*r*)”, or “The time derivative of the incremental energy (*dW*) absorbed by (dissipated in) an incremental mass (*dm*) contained in a volume element (*dV*)”. The relation expresses the *SAR* value

(11) $$SAR=\frac{d}{dt}\frac{\left(\Delta W\right)}{\left(\Delta m\right)}=\frac{d}{dt}\frac{\left(\Delta W\right)}{\left(\rho \Delta V\right)}=\frac{\sigma E^{2}}{\rho },$$

where Δ*W*—energy increment, Δ*m*-weight gain in tissue volume, *ρ*—tissue density *E*—effective value of electrical intensity fields in the tissue, *σ*—tissue conductivity. The *SAR* quantity specific absorbed rate is an important quantity used in non-ionizing radiation dosimetry. It is a measure of energy absorption, which can manifest itself in the form of heat, from which the internal fields in the tissue can be assessed, which can influence the internal processes in the living system. The three parameters’ definitions were reached: specific absorption rate, local peak *SAR*, and whole body-average *SAR*. The occurred absorption influences the parts of the body near the radiated device at frequencies from 100 kHz to 300 GHz. Therefore, the local peak *SAR* limit is considered the most critical value [10,11,12,13,14].

Study [14] evaluates the absorption in a user’s head of an EMF emitted by the Wi-Fi and/or Bluetooth module of a wearable small Internet of Things (IoT) electronic device (emitting EMF of up to 100 mW), to test the hypothesis that EMF has an insignificant influence on humans, and to compare the levels of such EMF absorption in various scenarios when using this device. The obtained results show up to 10 times higher values of *SAR* from the meandered inverted-F antenna (MIFA)-type antenna located in the headband, in comparison to its location on the helmet. Only wearable IoT devices (similar in construction and way of use to the investigated device) emitting at below 3 mW equivalent isotropically radiated power (EIRP) from Wi-Fi/Bluetooth communications modules may be considered environmentally insignificant EMF sources.

In another research, [15], data from the five largest monitoring networks were published and two extensive in situ measurement campaigns in different European countries were gathered and processed. Median electric field values for monitoring networks across various countries lay within 0.67–1.51 V/m. As evaluated from in situ measurements, the median electric field value across different microenvironments varied from 0.10 V/m to 1.42 V/m. The differences between networks were identified and mainly attributed to variations in population density. No significant trends in the temporal evolution of EMF levels were observed. The influences of parameters such as population density, type of microenvironment, and height of measurement on EMF levels were investigated.

The study [16] stated, based on the survey performed among medical practitioners, that high-frequency electromagnetic radiation has, according to their experience, an impact on human health, and it could cause diseases mainly of the neural system and negatively influence human reproduction functions.

Wi-Fi devices operated inside a metallic enclosure have been investigated in [17]. A motivation for this study was to investigate wave propagation inside an enclosed environment such as elevator, car, aircraft, and spacecraft. There are performances and safety concerns when the RF transmitters are used in the metallic enclosed environments. In this study the field distributions inside a confined room were investigated with multiple portable Wi-Fi devices. Computer simulations were performed using the rigorous computational electromagnetics (CEM). The method of moments (MoM) was used to model the mutual coupling among antennas. They show the possibility to maximize or minimize field intensity in a specific area by arranging the Wi-Fi devices as a function of the relative location and spacing in a calculated manner.

The results of studies [18], [13] and [19] also point out the potential radiation risks for people with cochlear implants in shielded spaces where they are exposed to EMF. This situation occurs during a telephone call in a railway carriage, affecting the person making the call as well as other persons in the vicinity. The resulting simulation values showed that the calculated maximum spatial average SAR values were approximately 1.57/1.19 times higher for 900 MHz exposure and 17 mm/22 mm head surface to PIFA antenna than the recommended maximum values set by the European limit SAR. They even determined the optimal distance between a mobile phone and a cochlear implant, 25 mm, in the environment of a railway vehicle.

In this study we decided to investigate the EMF inside shielded areas frequently used in everyday life such as lifts and personal vehicles.

## 2. Experimental Set-Up and Results

**Figure 3:**
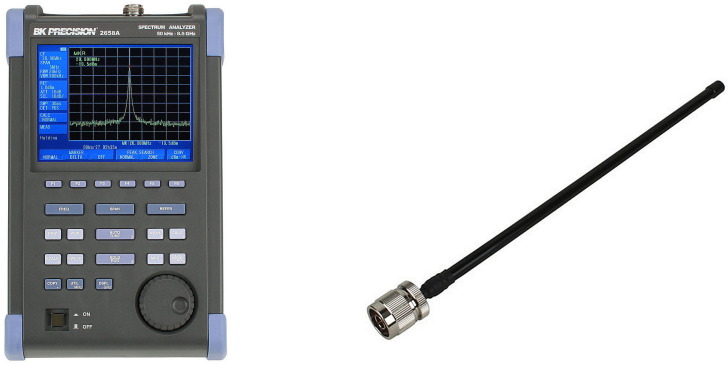
Used spectrum analyzer BK PRECISION 2650A and dipole antenna M40x.

**Figure 4:**
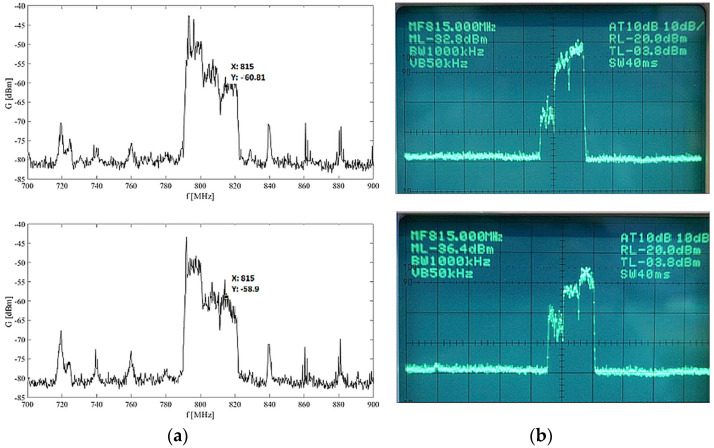
Example of calibration curves from both devices: digital spectral analyzer (**a**) and analog spectral analyzer (**b**).

For the measurement, the spectrum analyzer BK PRECISION 2650A was used. It is designed to measure the EMF in the frequency range 50 kHz–3.3 GHz, with B&K Precision 2650 dipole antennas in the following configurations: M401 (0.8 to 1 GHz), M402 (1.25 to 1.65 GHz), M403 (1.7 to 2.2 GHz), and M404 (2.25 to 2.65 GHz), Figure 3. Before the actual measurement, we verified the output from the BK PRECISION 2650A and the used antennas with SPECTRUM ANALYZER HM 5530 with broadband discone antenna, Figure 4. The wave impedances of utilized antennae are equal to 50 Ω. Figure 3 shows the device in use together with the antennas. The measured values were processed and displayed in the Matlab software. For displaying of spatial distributions of EM radiation with specific frequencies in space we used the color map displays. The spectrum analyzer was set in the regime that determined the maximum value for individual frequencies in the measured interval in 7 s. 

### 2.1. Case 1, Measurement Inside the Elevator Cabin

**Figure 5:**
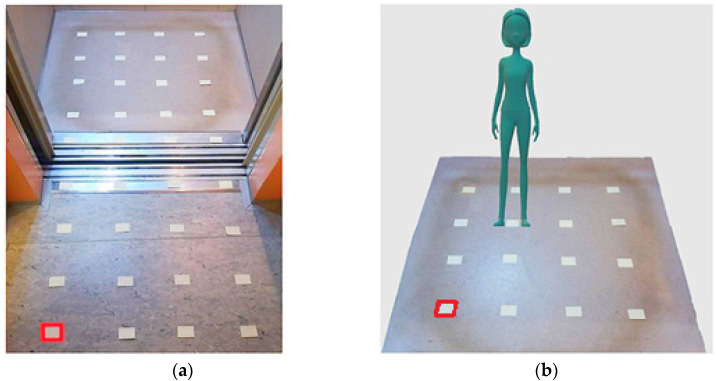
Measurement setup description: (**a**) representation of measurement points inside the empty elevator and in front of the elevator; (**b**) location of measurement points and position of the person on the phone in the elevator area.

The measurements in the elevator are carried in two ways—in an empty elevator, and inside an elevator with one “person” on the phone. The person is represented by the original phantom of human head with phone. In situation of empty and occupied elevator are all measurement points at a height of 150 cm from the floor level. When measuring the empty elevator, we also measure the field in the corridor, just in front of the elevator for comparison. The measurement started in the hall in the lower left corner highlighted in red, Figure 5a. After measuring the EM field values in the first row, the measurement continued in the second row again in the direction from left to right. Measurement points are 20 cm apart. In this way, 36 points were created in which we measured values of EM radiation.

**Figure 6:**
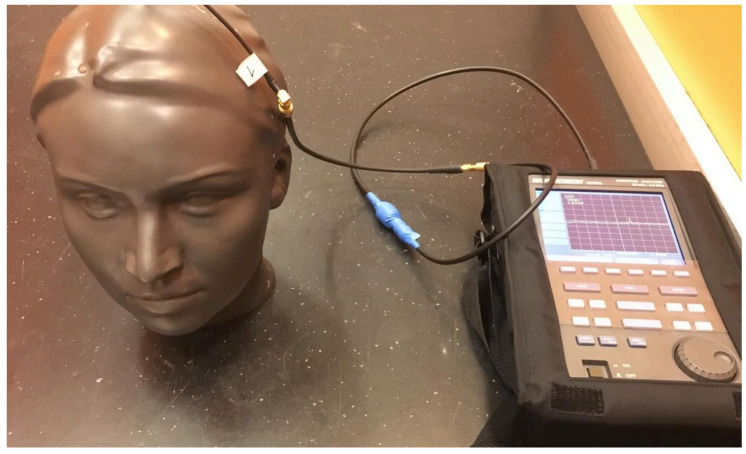
Human head phantom (made using rapid prototyping technology) for microwave EMF measurements usage.

Measurements of the EMF inside the human head phantom are carried out by the spectrum analyzer BK PRECISION 2650A with Tango 34 antenna with Quad Band 2G/GPRS. The phantom, Figure 6, represents a model of the human head constructed from two materials, using materials with properties resembling human tissue as much as possible. It is made of materials as similar as possible to the brain and bone tissue. The cover is made of polyurethane with additives to match the dielectric properties of bone tissue as closely as possible. The inside is filled with a solution that models the properties of brain tissue. A homogeneous solution is used to fill the phantom. This solution has the electrical properties of a brain tissue whose behavior is interesting for research. The solution allows free movement of the measuring probe in the volume of the phantom. Since the electrical properties of the solution are frequency dependent, it should be specially formulated for each frequency band to which the phantom is exposed. The values of conductivity and relative permittivity are calculated according to Equations (12) and (13) respectively, defined in the IEC/IEEE 62704-1:2017 standard [20]

(12) $$\sigma =0.805+0.00015\left(f\right)+4.12\cdot 10^{-8}{\left(f\right)}^{2}+2.87\cdot 10^{-11}{\left(f\right)}^{3},$$

(13) $$\varepsilon_{r}=46.52-0.006\left(f\right)+1.59\cdot 10^{-6}{\left(f\right)}^{2}-1.4\cdot 10^{-10}{\left(f\right)}^{3}.$$

The standard gives specific values of conductivity and relative permittivity for each frequency. Since the recipes for the ingredients used in the preparation of the solutions do not have exact values and inaccuracies also arise from the fact that each component has different properties, a 5% error rate is therefore given during measurement and testing.

**Figure 7:**
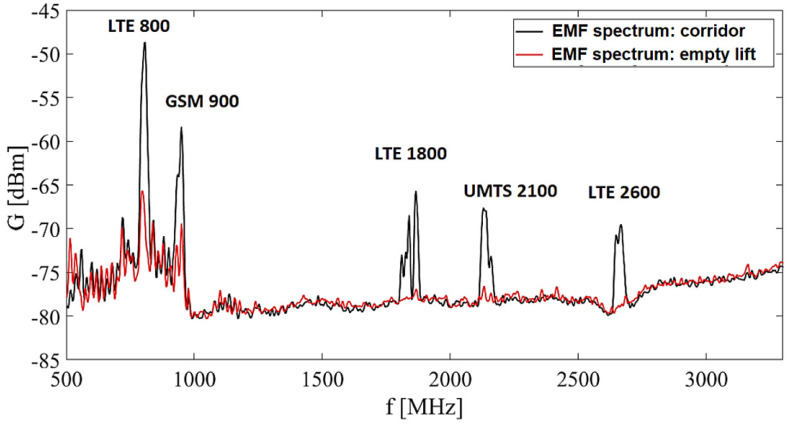
Experimental results: EM field received power spectrum in region of interest, individual sources.

Measurement started with the case of the empty elevator cabin and the area just in front of it to cover the open space in the vicinity. Figure 7 illustrates the record from the spectral analyzer from one measurement point in the corridor and one measurement point in the empty elevator.

It is possible to observe a significant decrease in the values of the EM field in the elevator (shielded space) compared to measurements in the corridor, Figure 7. For representation of the radiation distribution with specific frequencies in the investigated space are used color maps. To show graphically the distribution of EM radiation we choose the frequency with the highest amplitude in the given scope 500–3300 MHz interval.

**Figure 8:**
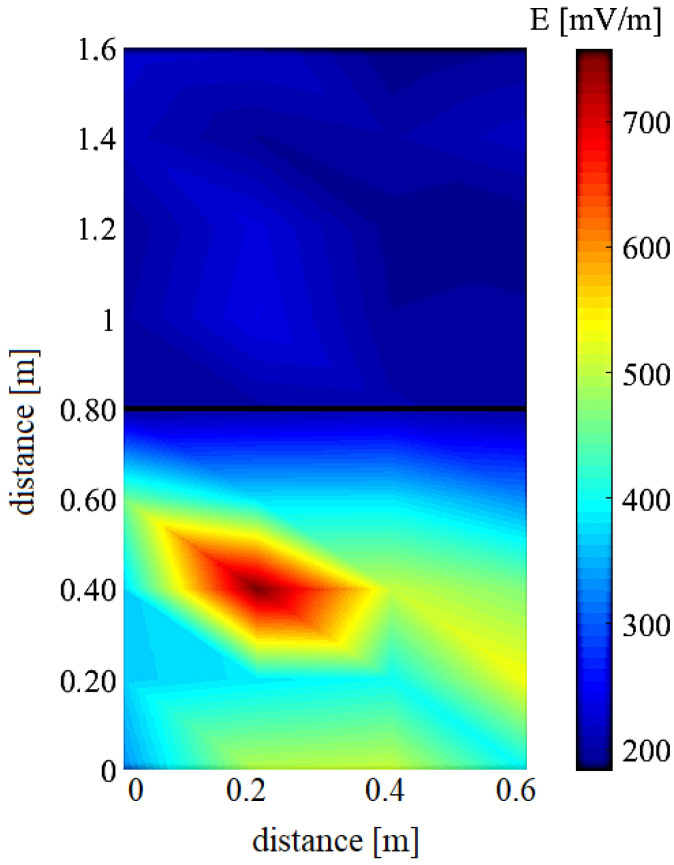
Experimental results: electric field strength spectrum in region of interest: *f* = 800 MHz. The line shows the border corridor/elevator.

Figure 8 shows the radiation distribution with a frequency of 800 MHz, Figure 8. We observe a significant decrease in radiation values in the elevator, which is composed of metal walls through which radiation does not penetrate to the same extent as in free space.

**Figure 9:**
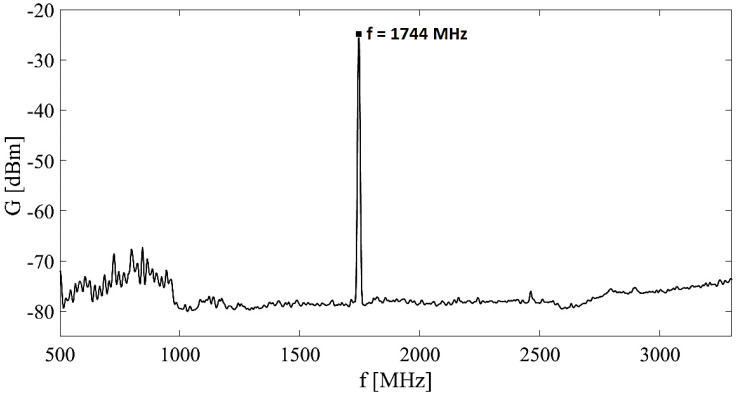
Experimental results: EM field received power spectrum in region of interest.

In the measurement representing the investigation in shielded area is a person is on the phone in the elevator. The network mode is WCDMA/GSM. The measurement started in the left corner, from left to right. The measurement points were 20 cm apart. The picture shows a representation of the position of the person during the measurement, Figure 9.

**Figure 10:**
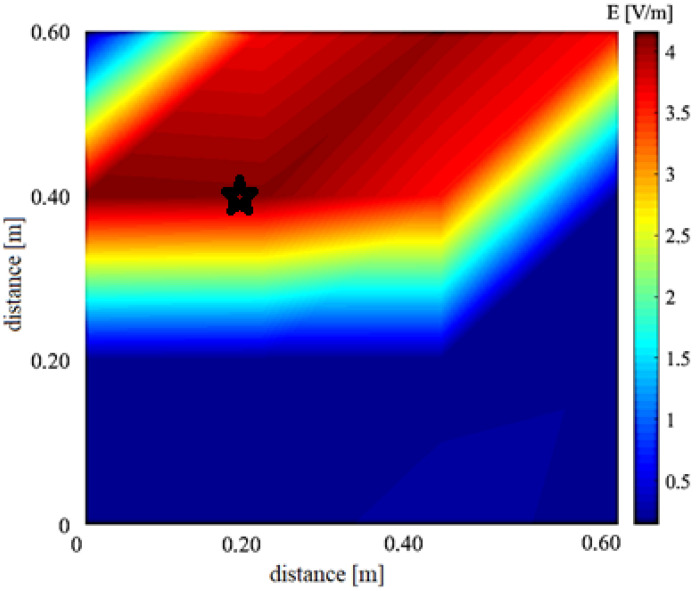
Experimental results: electric field strength spectrum in region of interest during one person on the phone: *f* = 1744 MHz.

From the recording from the spectrum analyzer, we can determine the frequency of the used mobile phone, which is 1744 MHz. Figure 10 shows the radiation distribution with a given frequency (in the interval 1700–1800 MHz). The highest value of E at the frequency of 1744 MHz is at the position indicated by a star, which corresponds to the position of the person in space.

Measurement inside the phantom is performed without phone and using the phone placed to the left ear in open space in front of the elevator and inside the cabin. The phantom is placed in the position of the person inside the elevator cabin, Figure 5b. Measured values of electric field strength for both open and shielded space measurements inside the phantom are shown in Table 2.

Measurement with a human head phantom in a shielded environment shows that the value of electrical intensity for a 6-min interval was not exceeded. A call in a shielded environment could, especially with more than one RF source may cause a rise in values even exceeding the limit values [13,17,18]. It highly depends on the type of EMF source and on what frequency in the mobile band the phone connects to the base station. Therefore, it is advisable to use hands-free to avoid possible greater exposure to EMF during longer calls.

### 2.2. Case 2, Measurement in the Vehicle

**Figure 11:**
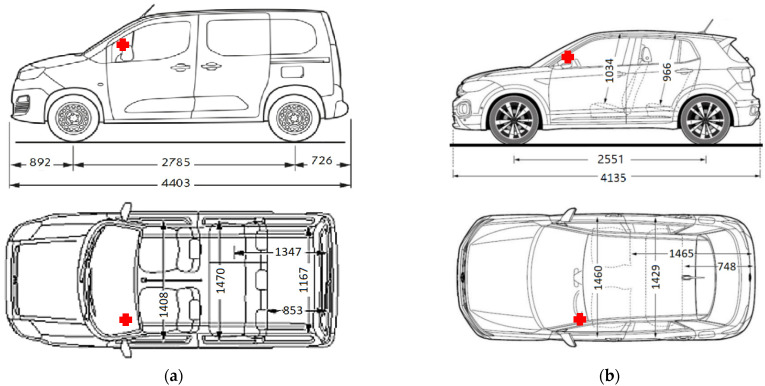
Experimental setup: measurement point (red cross) inside the car (**a**) CAR A, and (**b**) CAR B.

The measurements are carried out in a typical urban setting during the day. Two distinct car models, CAR A (the year 2015) and CAR B (the year 2019) with embedded wireless technologies were used for the measurement, Figure 11. For connection of the phone to the phone interface in the car, CAR A uses Bluetooth technology. In the case of CAR B, in addition to hands free more wireless technologies are used, for purposes other than making phone calls. If a phone has already been connected to the automobile, some phones are automatically detected and connected when the ignition is turned on. Data are transferred using one of the Bluetooth profiles when the phone is connected to the phone interface. The Message-Access-Profile (MAP) and Phone-Book-Access-Profile (PBAP) are automatically connected to the Hands-free Profile when telephone activation occurs (HFP). The Advanced Audio Distribution Protocol (A2DP) and Audio/Video Remote Control Protocol (AVRCP) will immediately connect to the phone even if A2DP is already active. The focus of measurement is on the drivers’ seat because the driver spends the most time in the vehicle using the available technologies, Figure 11.

**Figure 12:**
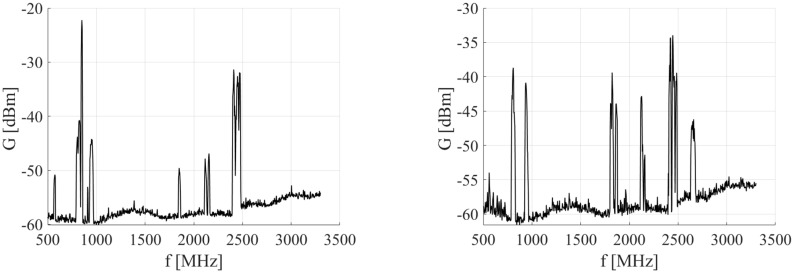

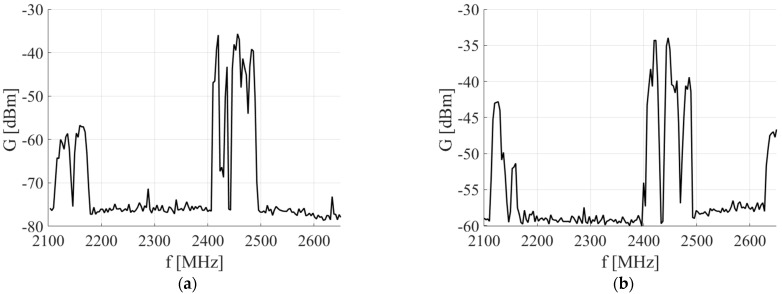
Example of EMF received power of whole spectrum (upper graph) and spectrum in measured frequency band (lower graph) (**a**) CAR A, and (**b**) CAR B.

Examples of measured EMF whole spectrum and in inspected frequency band are shown in Figure 12 for both measured cars. The results from each measurement are computed, and the absolute value of field strength above the driver’s seat within the car is evaluated. Maximum obtained values of field strength E for measured frequency range from 2100–2500 MHz are shown in Table 3. Although it depends on the frequency range and the type of car, the field strength levels in CAR B are relatively low. The highest value of field strength was in case without phone call recorded by CAR A at the frequency 2144 MHz, while CAR B’s maximum value occurred at the frequency 2139 MHz. During the ongoing phone call are the values in both measured bands higher in CAR A. CAR B has, during the call, the highest values only in frequency range 2400–2500 MHz.

For both measured cars in case of ongoing phone calls the values are higher. The value in both cars varies significantly, and in the older car are in most measured cases higher than in the newer car. Increase of the electric field strength levels from the switched-off phone state to the ongoing call in is observable especially in Bluetooth bandwidth. However, limits of electric field strength for the observed frequencies are set to significantly higher values than the measured maximum values.

## 3. Conclusions

This article dealt with the measurements of the EM field in the microwave frequency band in shielded areas. A dipole antenna was used to perform the experiments, while a discone antenna was also used to verify the measurement results and calibrate the instruments. The assessment of the degree of possible EMF influence in the microwave band was carried out given the current legislation in force in the EU. Hygienic limits set maximum permissible EMF values, converted to given tissue parameters, but they do not include situations where EMF sources are in a shielded environment (for example, underground parking garages, garages, cellars, personal elevators, etc.). This study showed that the values are significantly elevated in the premises in question. At the same time, under certain circumstances (for example, several people talking on the phone in an elevator simultaneously, etc.) it can potentially affect the proper function of various implantable electronic devices (pacemaker, insulin pump, etc.) [13,18,21].

Measurements in shielded spaces such as elevators and car cabins show the raised values of RF fields when using communication devices, even car-embedded hands-free. It is recommended to avoid using the phone in a shielded environment or vehicles. The effects of exposure to electromagnetic fields depend on the human health constitution and qualification.

The directives and standards limiting the exposure of EMF are electric field strength limits for the frequency range up to 1800 MHz for “Public” 58 V/m and “Occupational” 127 V/m and for the frequency range up to 2100 MHz for “Public” 61 V/m and “Occupational” 137 V/m. The electric field strength in shielded space is much lower in our measurements than in exposure limits. Nevertheless, it is advisable to approach the use of mobile devices, especially in shielded areas, with caution.

## Figures and Tables

**Table 1:** ICNIRP steady-state operational-thresholds and basic restrictions for occupational and public exposures [9].

	Core Body Temperature	Local Temperature (Head, Torso)	Local Temperature (Limbs)	Local Temperature (Head, Torso, Limbs)	
Frequency range	100 kHz–300 GHz	100 kHz–6 GHz	100 kHz–6 GHz	>6–300 GHz	30–300 GHz
Operational threshold	+1°	+2°	+5°	+5°	+5°
Spatial averaging	WBA	10 g	10 g	4 cm^2^	1 cm^2^
Temporal averaging	30 min	6 min	6 min	6 min	6 min
EMF threshold	4 W/kg	20 W/kg	40 W/kg	200 W/m^2^	400 W/m^2^
Reduction factor	10	2	2	2	2
Occupational	0.4 W/kg	10 W/kg	20 W/kg	100 W/m^2^	200 W/m^2^
Reduction factor	50	10	10	10	10
Public	0.08 W/kg	2 W/kg	4 W/kg	20 W/m^2^	40 W/m^2^

**Table 2:** Maximum values of electric field strength measured in head phantom for frequency band 1700–1800 MHz.

Position	Without Call E [mV/m]	Ongoing Call E [mV/m]
Open space	1.0	6.8
Elevator cabin	0.89	12.2

**Table 3:** Maximum values of electric field strength measured in CAR A and B.

Frequency Band [MHz]	CAR	Without Call E [mV/m]	Ongoing Call E [mV/m]
2100–2150	A	236.85	254.96
2400–2500	A	117.00	199.38
2100–2150	B	41.83	55.26
2400–2500	B	8.2	218.10

## Data Availability

The data presented in this study are available on request from the corresponding author.
